# ID 307 - Avaliação do Perfil de Prescrição e Troca dos Medicamentos Modificadores do Curso da Doença Biológicos e Sintéticos Alvo-Específicos para o Tratamento da Artrite Psoríaca no Sistema Único de Saúde

**DOI:** 10.5327/2237-9622.2025.v34s1.307

**Published:** 2025-11-25

**Authors:** Lívia Silva Nassif, Bárbara Rodrigues Alvernaz dos Santos, Marina Morgado Garcia, Flávia Alves, Grazielle Dias, Augusto Afonso Guerra, Francisco de Assis Acurcio†, Juliana Alvares-Teodoro

**Keywords:** artrite psoríaca, medicamentos modificadores do curso da doença, Sistema Único de Saúde (SUS)

## Abstract

**Introdução:**

A artrite psoríaca (AP) é uma doença inflamatória articular associada à psoríase, pertencente ao grupo das espondiloartrites. Estima-se que a incidência global da doença seja de aproximadamente 83/100.000 pessoas-ano. A AP apresenta manifestações clínicas heterogêneas, incluindo acometimentos dermatológicos e musculoesqueléticos, que afetam a qualidade de vida desses pacientes. Conforme preconizado pelo Protocolo Clínico e Diretrizes Terapêuticas (PCDT) da AP, a conduta terapêutica inicial inclui o uso de glicocorticoides, imunossupressores e anti-inflamatórios não esteroidais para controle sintomático, e o uso de medicamentos modificadores do curso da doença sintéticos (MMCDs) para minimizar a atividade da doença. Caso haja falha em pelo menos dois esquemas terapêuticos com MMCDs, utilizam-se os medicamentos modificadores do curso da doença biológicos (MMCDbio) ou sintéticos alvo-específicos (MMCDsae). Apesar das inúmeras linhas de tratamento, a AP e as demais condições reumáticas continuam a ser um problema de saúde pública. O objetivo deste trabalho é descrever o perfil de prescrição e troca dos MMCDbio e/ou MMCDsae em pacientes diagnosticados com AP tratados em Minas Gerais, Brasil.

**Método:**

Foram coletados, por meio do Sistema Integrado de Gerenciamento da Assistência Farmacêutica de Minas Gerais, dados dos processos deferidos de solicitação de MMCDbio e/ou MMCDsae ao Componente Especializado da Assistência Farmacêutica (Ceaf-MG/SUS) para AP. O período de análise compreendeu de janeiro de 2018 a dezembro de 2023, com base na data do parecer do analista. Os dados foram organizados em planilha do Excel. O número do Cartão Nacional de Saúde foi utilizado para identificar os pacientes e ordenar as trocas medicamentosas. O tempo de uso foi calculado subtraindo-se a data da primeira dispensação da data da última registrada. Foram excluídos da análise os pacientes que não tinham registro de dispensação.

**Resultados:**

Dos 2.570 pacientes incluídos na análise final, 67,8% (1742) foram diagnosticados com o CID M07.3. A idade média no momento da primeira dispensação foi de 51 anos. O primeiro MMCDbio mais prescrito foi o adalimumabe (39,7%), seguido por golimumabe (18,8%), secuquinumabe (17,5%) e infliximabe (13,2%). O tempo médio de uso do primeiro medicamento prescrito foi de 28,1 meses, com tempo mínimo de 1 mês e máximo de 74 meses. Dos 2.570 pacientes, 299 (11,6%) necessitaram trocar de medicamento e, nesses casos, o etanercepte teve o maior percentual de trocas (19,0%), seguido por tofacitinibe (15,4%), infliximabe (13,9%) e adalimumabe (12,4%). Após a troca, os medicamentos mais prescritos como segunda opção terapêutica foram o secuquinumabe (54,5%), adalimumabe (13,1%) e golimumabe (8,4%). O tempo médio de uso do segundo medicamento foi de 20,2 meses, variando de 1 a 60 meses. Desse grupo, 8,7% (26/299) necessitaram trocar o segundo biológico prescrito.

**Conclusão:**

A disponibilidade de MMCDbio e/ou MMCDsae no SUS oferece esperança de regressão da atividade da doença e de melhora da qualidade de vida dos pacientes que falham na primeira linha de tratamento. Este estudo reforça a importância dos dados administrativos do Sistema Único de Saúde (SUS) para análises farmacoepidemiológicas. Além disso, entender o perfil de prescrição desses medicamentos é essencial para guiar a seleção, a programação e a aquisição de medicamentos para a AP no País.

